# Arterial spin labeling magnetic resonance imaging quantifies tissue perfusion around foot ulcers

**DOI:** 10.1016/j.jvscit.2022.09.015

**Published:** 2022-10-03

**Authors:** Joe Luis Pantoja, Fadil Ali, Donald T. Baril, Steven M. Farley, Scott Boynton, J. Paul Finn, Peng Hu, Peter F. Lawrence

**Affiliations:** a Division of Vascular and Endovascular Surgery, David Geffen School of Medicine, University of California Los Angeles, Los Angeles, CA; b Department of Radiology, David Geffen School of Medicine, University of California Los Angeles, Los Angeles, CA; c Division of Vascular Surgery, Cedars-Sinai Medical Center, Los Angeles, CA

**Keywords:** Arterial spin labeling, Diabetic foot ulcer, Perfusion

## Abstract

**Objective:**

Tools that quantify tissue perfusion of the foot are deficient, contributing to the uncertainty in predicting ulcer healing potential. This pilot study aims to quantify peri-wound foot perfusion at various tissue depths using a novel application of pseudo-continuous arterial spin labeling magnetic resonance imaging.

**Methods:**

Ten diabetic patients with neuropathic wounds and 20 healthy volunteers without wounds were recruited. Wounds were graded according to the Wound, Ischemia, Foot Infection (WIfI) system. All subjects underwent a noncontrasted ASL MRI of the foot for perfusion measurements. For healthy volunteers, perfusion was compared at rest and during sustained toe flexion between four regions: lateral plantar, medial plantar, lateral calcaneal, and medial calcaneal. Evaluations of diabetic volunteers compared perfusion between four zones: wound, near border, far border, and remote. Remote zone perfusion in diabetics was compared with perfusion in the plantar foot of healthy volunteers.

**Results:**

There were 11 wounds, which were located over the metatarsal heads in five, the stump of a transmetatarsal amputation in three, the heel in two, and the mid foot in one. The median WIfI stage was 2. One patient had a WIfI ischemia grade of 1; the remaining patients’ grades were 0. The mean ankle-brachial index was 1.0 ± 0.3. There were two patients with a WIfI foot infection grade of 1; the remaining patients’ grades were 0. In healthy volunteers, plantar foot perfusion with sustained toe flexion was 43.9 ± 1.7 mL/100g/min and significantly higher than perfusion at rest (27.3 ± 2.7 mL/100g/min; *P* < .001). In diabetic patients, perfusion at the wound, near border, far border, and remote regions was 96.1 ± 10.7, 92.7 ± 9.4, 73.4 ± 8.2, and 62.8 ± 2.7 mL/100g/min. Although this perfusion pattern persisted throughout the depth of the wound, perfusion decreased with tissue depth. In the near border, perfusion at 20% of the wound depth was 124.0 ± 35.6 mL/100g/min and 69.9 ± 10.1 mL/100g/min at 100% (*P* = .006). Lastly, remote perfusion in diabetics was 2.3 times the plantar perfusion in healthy volunteers (27.3 ± 2.7 mL/100g/min; *P* < .001).

**Conclusions:**

The pattern of resting tissue perfusion around nonischemic diabetic foot ulcers was successfully quantified with arterial spin labeling magnetic resonance imaging. Diabetic patients with wounds were hyperemic compared with healthy volunteers. There was a 1.5-fold increase in peri-wound tissue perfusion relative to the rest of the foot. This study is the first step in developing a tool to assess the perfusion deficit in ischemic wounds.

Foot ulcers are a prevalent source of morbidity in diabetic patients and may lead to major amputation. Patients with diabetes have a 25% risk of developing a foot ulcer over their lifetime, leading to major amputation in 17% of them by 1 year.^1^, ^2^, ^3^, ^4^, ^5^ Although neuropathy is the dominant etiology of foot wounds in the diabetic population, ischemia impacts 65% of these wounds.^6^ A key factor in determining the healing potential of these wounds is the perfusion deficit in the limb. The physiologic assessment of limb perfusion is now a recommended standard practice for evaluation of patients with chronic limb-threatening ischemia.^7^ Yet the tools to quantify limb perfusion are deficient.

The current tools to assess perfusion are either indirect or qualitative and do not provide a quantitative assessment of perfusion. The use of indirect tools like ankle-brachial index and toe-brachial index are widespread and have been included in recent disease grading classification systems.^8^^,^^9^ However, these tools are limited by medial calcinosis and do not provide a localized assessment around the wound.^10^ Transcutaneous oxygen measurement is another widely used tool whose sensitivity and specificity for predicting wound healing in the diabetic population is higher than the aforementioned indirect tools.^11^ However, this tool provides averaged data over large regions of the limb and is limited by tissue factors like edema and vasoconstriction.^12^ More recent qualitative methods including hyperspectral imaging and indocyanine green angiography, can detail local tissue perfusion throughout the foot.^13^, ^14^, ^15^ However, these tools limit their assessment to a superficial level and do not provide information on deeper tissue layers including muscle.

Arterial spin labeling (ASL) is a noninvasive magnetic resonance imaging (MRI) technique that quantifies tissue perfusion at various tissue depths, without intravenous contrast. Clinically, it has been used in neuroimaging to quantify brain tissue perfusion.^16^^,^^17^ Briefly, it tags protons in blood with radiofrequency pulses upstream from the tissue of interest. Images of the tissue of interest are acquired before and after perfusion with this labeled blood. Their subtraction yields a spatial map of perfusion throughout the imaged tissue. This has been applied to skeletal muscle in the extremities and, more recently, to the diabetic foot.^18^, ^19^, ^20^, ^21^

The objective of this study is to investigate the novel application of ASL to assess foot perfusion in both healthy volunteers without wounds and diabetic volunteers with foot ulcers. This pilot study aims to quantify peri-wound foot perfusion at various tissue depths and compare this with foot perfusion in healthy volunteers.

## Methods

### Study volunteers

To conduct this pilot study, 20 healthy volunteers were recruited from a university health campus and 10 diabetic volunteers were recruited from a university-affiliated wound care center. This study included healthy volunteers for two reasons: to measure perfusion in the healthy non-wounded foot and ensure our imaging protocol was able to detect changes in perfusion while volunteers performed maneuvers known to increase perfusion in the foot. All volunteers were required to be greater than 18 years old and able to undergo an MRI study. Additionally, diabetic volunteers were required to have an active foot wound, formally diagnosed diabetes mellitus via hemoglobin A1c or serial blood glucose examinations, and an objective physiologic assessment of macrovascular peripheral arterial disease with duplex ultrasound, ankle-brachial index, or toe-brachial index. Exclusion criteria for the healthy volunteers included structural or functional heart disease, diabetes, history of peripheral arterial disease, claudication or rest pain in any extremity, arterial injuries of the lower extremities, active tobacco use, or an abnormal ankle-brachial index (less than 0.9). This study was approved by the Institutional Review Board of the University of California Los Angeles. Written consent was obtained from all volunteers prior to participation in the study.

### Study design

After obtaining consent and screening volunteers, the ankle-brachial index was measured in healthy volunteers. Because the diabetic participants already had objective assessment of macrovascular disease completed in the wound care clinic with either ankle-brachial index or duplex ultrasound, an additional measurement of the ankle-brachial index was not measured at the time of the study. Although toe-brachial index is a standard measure of assessment especially in diabetic patients, the 10 volunteers with wounds in this study did not need toe-brachial indices to assess the presence of peripheral arterial disease as the ankle-brachial index and/or duplex ultrasound was sufficient. Next the participants were positioned supine on the MRI table with the foot of interest in a foot coil. All volunteers were allowed to rest for 5 minutes on the MRI table prior to commencing imaging. For healthy volunteers, the dominant foot was imaged, whereas in diabetic volunteers, the foot with the wound was imaged. All imaging was performed on a 3.0T Siemens Magnetom Skyra MR system (Siemens Healthineers, Malvern, PA). This MRI system is the same type of system used for a variety of clinical applications. No hardware modifications or specialty equipment is needed to implement this imaging protocol. Scout images were initially obtained in the axial and sagittal planes. Then a high resolution T2-weighted image was obtained throughout the foot to discern soft tissue anatomy including wound edges. Finally, the ASL sequence was performed using the same slice number and thickness. The ASL images were registered to the T2-weighted image, providing an anatomically aligned perfusion map. Healthy volunteers underwent a second round of imaging during sustained toe flexion. This imaging sequence was used to confirm the ability to detect changes in perfusion in an expected location in the foot. They were asked to plantarflex their toes such that they reduced the distance between the metatarsal head and the heel. This published method reduces the medial longitudinal angle of the foot and activates the intrinsic muscles of the foot, especially the abductor hallucis muscle.^22^^,^^23^ The exercise was demonstrated, and volunteers were allowed to practice. The volunteer’s foot was once again placed in the foot coil. They were asked to flex their toes as far as possible and hold the position throughout the duration of the study. Although they were asked to exert maximal force, there was no measure of the effort they exerted.

### Arterial spin labeling technique

We specifically used pseudo-continuous ASL to measure tissue perfusion.^24^ Briefly, ASL administers two acquisitions: control and tagging. Prior to either acquisition, an inversion process is applied upstream to the tissue of interest. For pseudo-continuous ASL, this inversion is only sensitive to flowing spins. The inversion is split into a series of low flip angle Hanning-shaped radiofrequency pulses, which have a lower energy deposition compared with a continuous adiabatic inversion. The tagged and control acquisitions are distinguished by the effects of these radiofrequency pulses on the flow spins. In the control acquisitions, the radiofrequency pulses are applied such that there is a zero net effect on spins. However, in tagged acquisitions, the radiofrequency pulses cause a preferential inversion along the arterial flow spins. After tagging, the inverted spins enter the capillary bed and exchange with the water molecules in the tissue. The inverted spins limit the amount of excitable water molecules in the tissue of interest. Therefore, the signal difference between the control and tagged images is proportional to perfusion.

### Parameters

Our pseudo-continuous ASL acquisitions were based on a 3D Turbo Gradient Spin Echo’s pulse sequence. This was employed with 10 spin echo trains, a 63 echo-planar factor, a 38.44 ms effective echo time, 4300 ms repetition time, a fat saturation pulse, and an 1800 ms inversion time. We had an in-plane 192 × 192 mm field of view with a corresponding 64 × 64 in-plane matrix. A single slab was sampled which featured eight 3-mm slices parallel to the plantar foot or plane of the wound. With 30 averages, our scan was achieved in 8:40 minutes per foot.

### Perfusion maps

We applied Flow Encoding Arterial Spin Tagging (FEAST) to measure the blood transit time from the tagging plane to the tissue.^25^ A traditional ASL signal can be described as:

$$\Delta M=\frac{{2M}_{0}f\alpha }{{\lambda R}_{1a}}\left[\exp\left(-wR_{1a}\right)-\exp\left(-\left(\tau +w\right)R_{1a}\right)\right)\;\left[1\right]$$

where τ is the labeling pulse duration, $R_{1a}$ is the longitudinal recovery rate of the blood, $M_{0}$ is calf equilibrium magnetization, w describes the delay time, *f* is the tissue blood flow, λ is the blood-tissue water partition coefficient, and α is the tagging efficiency. Modified to get a more accurate measure, the image acquisition was interleaved between tagging and control pulses. Flow encoding gradients were placed before the readouts along the slice-select’s duration. The gradients were 0 for odd pair scans and 2.1 G/cm for even pairs. This nonzero value spoiled the vascular signal, leaving only the tissue signal contributing to the ASL data:

$$\Delta M^{\prime }=\frac{{2M}_{0}f\alpha }{{\lambda R}_{1a}}M_{0}\left[\exp\left(-{\delta R}_{1a}\right)-\exp\left(-\left(\tau +w\right)R_{1a}\right)\right)\;\left[2\right]$$

where δ is the blood transit time. The assumed parameters values were 2 seconds delay time, 1.5 seconds label time, 0.8 tagging efficiency, 0.83 seconds^-1^ longitudinal recovery rate, and a 0.8 g/mL blood-tissue water partition coefficient.^25^

We would subtract the control image from the label image for each voxel (*ΔM'*), and then solve for the tissue flow with the parameters mentioned above.

### Outcome measurements and data analysis

**Fig 1:**
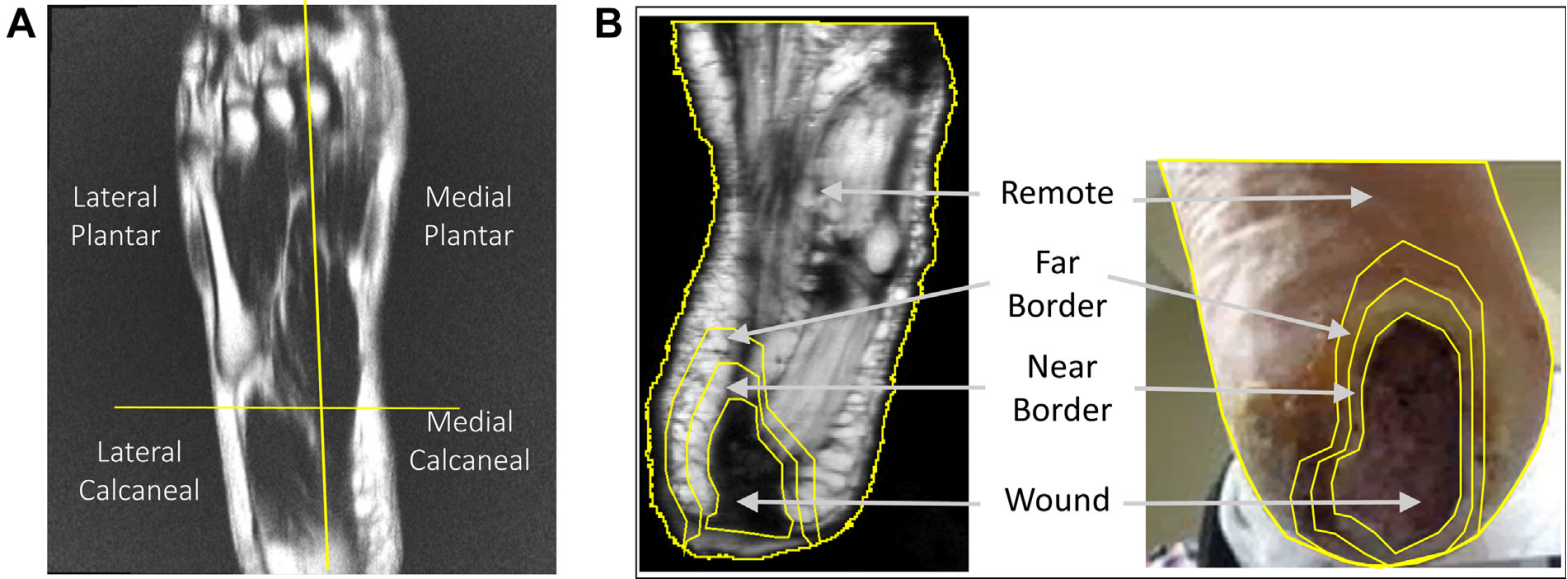
The plantar foot of healthy volunteers **(A)** was segmented into the medial and lateral plantar and calcaneal regions while that of the diabetic foot **(B)** was segmented into the following peri-wound region: wound, near border, far border, and remote zones.

For diabetic patients, the wound size, presence of infection, and vascular assessment were gathered during the pre-scan exam or from the electronic chart. Using this data, the Wound, Ischemia, and Foot Infection (WIfI) grades of the wounded limbs were calculated according to previously published guidelines.^6^ After imaging, a perfusion map was generated using custom image analysis software (MATLAB, MathWorks, Natick, MA) that implemented the FEAST perfusion calculation. The custom imaging analysis algorithm was developed by the group and could be run on any computer with MATLAB. A perfusion map was generated for each imaged ASL slice, which showed the perfusion for each voxel in an image. Then a region of interest (ROI) was created around the foot at each slice on the T2-weighted images. The foot ROI for healthy volunteers was subdivided into lateral and medial plantar and calcaneal regions (Fig 1, *A*). The medial and lateral division was between the second and third metatarsal, whereas the plantar and calcaneal division was at the transverse tarsal joint. For the volunteers with wounds, the ROI (unique to each image slice) was sectioned into 4 areas: wound, near border zone, far border zone, and remote zone. The near border zone is an area surrounding the wound that is 125% of the wound area, whereas the far border zone is 150%. Both were centered around the wound (Fig 1, *B*). The remote zone is the tissue outside the far border zone. When comparing diabetic and healthy volunteers, the entire ROI of the plantar foot in healthy volunteers corresponded to the remote zone in volunteers with wounds. In healthy volunteers, regional perfusion at rest and during sustained toe flexion were compared. The average remote zone perfusion in diabetic volunteers was compared with that in healthy volunteers at rest. Lastly, we compared the average perfusion amongst peri-wound zones.

### Data analysis

All perfusion values are expressed as mL/100g of tissue/min. Descriptive statistics are expressed as mean ± standard error for quantitative variables and frequency (percentiles) for categorical variables. WIfI grades are expressed as median (range). Continuous variables were analyzed with regression using generalized linear mixed models, which can perform differences in means analysis for two or more groups while accounting for both fixed and random effects. In each analysis, the individual was held as a random variable. The R Statistical package was used to perform all statistical analysis (Version 3.5.1, R Core Team, Vienna, Austria).^26^

## Results

### Volunteer demographics and wound characteristics

The diabetic group of patients were older and had higher rates of smoking compared with the healthy volunteer group (Table I). The ABI between the two groups were similar (1.0 ± 0.3 vs 1.1 ± 0.03). None of the volunteers with wounds had toe-brachial indices measured in the limb with the wound. Three patients in the diabetic group had undergone a transmetatarsal amputation in the past for infection. The diabetic group had a median WIfI stage of 2 (range, 1-3). The median wound grade was 2 (range, 1-3), ischemia grade was 0 (range, 0-1), and foot infection grade was 0 (range, 0-1). There was one volunteer with an ischemia grade of 1; the others had grades of 0. There were three volunteers with a foot infection grade of 1; the others had grades of 0. The mean duration of the wounds was 14.1 ± 3.0 months, and the size was 4.3 ± 1.9 cm^2^ at the time of the study. Wounds were located at the metatarsal head (n = 5), heel (n = 2), and at the transmetatarsal amputation stump (n = 3).

**Table I:** Volunteer demographics and wound characteristics

Characteristics	Diabetic wounds (n = 11)	Healthy volunteers (n = 20)
Male sex	11 (100%)	14 (70%)
Age, years	60.1 ± 3.1	26.8 ± 0.8
Diabetes mellitus	11 (100%)	0 (0%)
Current smoking	7 (64%)	0 (0%)
Ankle-brachial index	1.0 ± 0.3	1.1 ± 0.03
Wound duration, months	14.1 ± 3.0	–
Wound location		
Metatarsal head, plantar	5 (45%)	–
Mid foot, plantar	1 (9%)	–
Transmetatarsal amputation stump	3 (27%)	–
Heel	2 (18%)	–
Wound area, cm^2^	4.3 ± 1.9	–
WIfI classification score		
Wound	2 (1-3)	–
Ischemia	0 (0-1)	–
Foot infection	0 (0-1)	–

*WIfI*, Wound, Ischemia, and foot Infection.

Data are presented as number (%), mean (standard error), or median (range).

### Perfusion in the plantar foot

**Fig 2:**
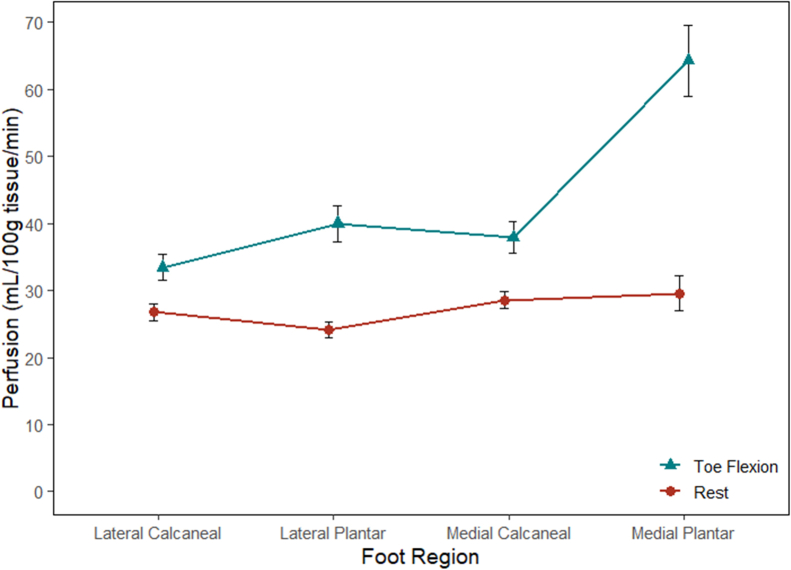
Regional foot perfusion in healthy volunteers during rest and sustained toe flexion.

**Fig 3:**
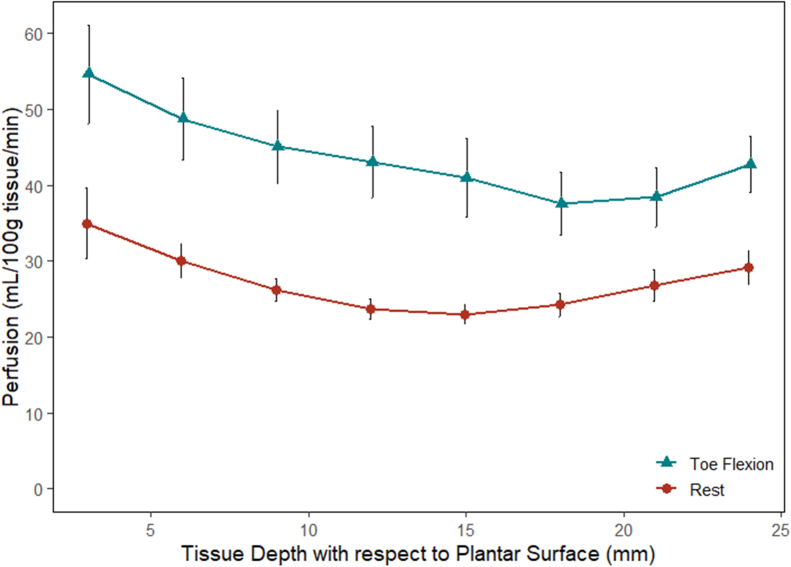
Plantar foot perfusion at various depths in healthy volunteers during rest and sustained toe flexion.

In healthy volunteers, the average tissue perfusion in the plantar aspect of the foot at rest was 27.3 ± 2.7 mL/100g/min. There was no difference in perfusion between the various foot regions at rest (Fig 2). During sustained toe flexion, the average foot perfusion was 43.9 ± 1.7 mL/100g/min and was significantly different compared with rest (*P* < .001). The most substantial increase in perfusion occurred over the medial plantar foot during toe flexion compared with the resting state (64.3 ± 5.3 vs 29.6 ± 2.6 mL/100g/min; *P* < .001). This increase in perfusion was detected throughout the thickness of the foot and was most prominent superficially (Fig 3). The perfusion in the healthy plantar foot tissue was significantly higher in diabetics with wounds compared with healthy volunteers at rest (62.8 ± 2.7 vs 27.3 ± 2.7 mL/100g/min; *P* < .001).

### Peri-wound perfusion

**Fig 4:**
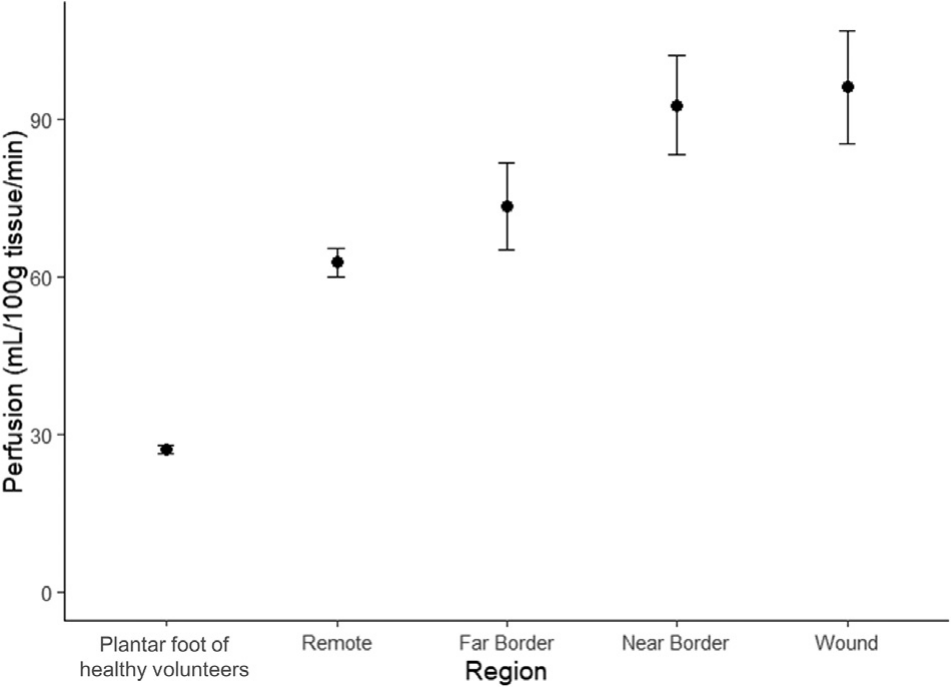
Regional foot perfusion around nonischemic diabetic foot ulcers.

**Fig 5:**
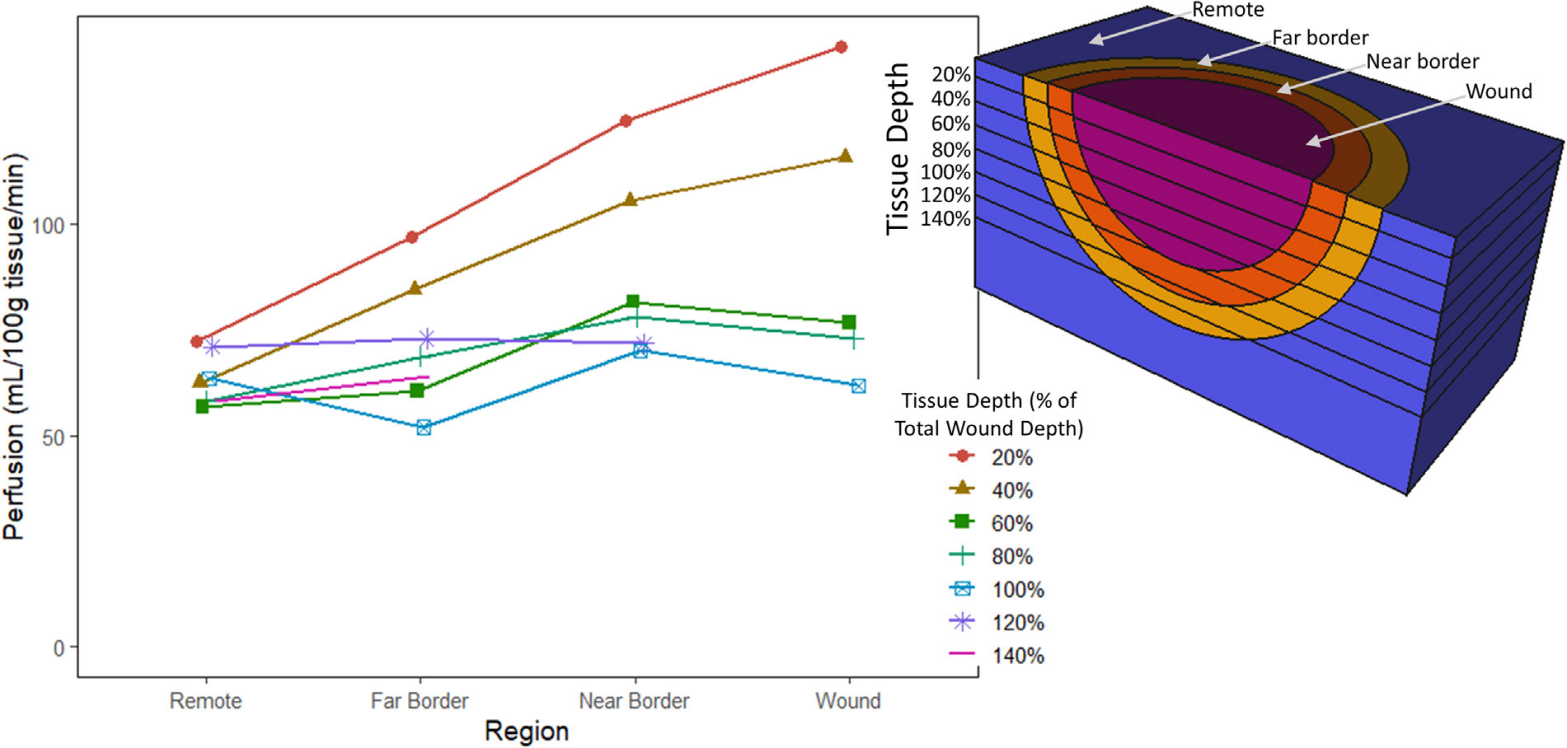
Regional peri-wound foot perfusion in nonischemic diabetic foot ulcers at various depths with respect to the total wound depth.

There was a distinct perfusion gradient around the wounds in diabetic volunteers (Fig 4). Perfusion in the wound (96.1 ± 10.7 mL/100g/min) and the near border zone (92.7 ± 9.4 mL/100g/min) was significantly higher than that in the remote zone tissue (62.8 ± 2.7 mL/100g/min; *P* < .001 and *P* = .002, respectively). There was no significant difference between the wound and near border zone perfusion (*P* = .984). Perfusion at the far border zone (73.4 ± 8.2 mL/100g/min) was higher than the remote zone but not significantly (*P* = .549). In Fig 5, the peri-wound perfusion pattern at various tissue depths can be seen. Tissue perfusion in the wound and the near border zone are highest superficially. There was a marked decrease in perfusion at 60% of the wound depth in the wound region and near border. Furthermore, the difference in perfusion between peri-wound zones and the remote zone are not significant at greater tissue depths (Table II). When comparing the wound and remote regions, the variation in perfusion at 60% is not significantly different (76.6 ± 23.7 vs 56.7 ± 6.7 mL/100g/min; *P* = .403).

**Table II:** Regional peri-wound perfusion at various wound depths with comparisons between the wound and remote regions at a given depth (bottom row) and at 20% and 100% of the wound depth at a given region (right column)

	Depth					*P*-value (20% vs 100%)
	20%	40%	60%	80%	100%	
Region						
Wound	141.7 ± 33	115.5 ± 23.6	76.6 ± 23.7	72.6 ± 14.6	61.7 ± 10.3	.015
Near border	124 ± 35.6	105.2 ± 19	81.5 ± 15.8	77.9 ± 16.2	69.9 ± 10.1	.006
Far border	96.6 ± 27	84.4 ± 17.5	60.4 ± 16.8	68.5 ± 16.7	51.8 ± 9.4	.028
Remote	72.1 ± 6.1	62.3 ± 5.5	56.7 ± 6.7	58.1 ± 4.5	63.4 ± 7.5	.27
*P*-value (wound vs remote)	.0116	.0115	.403	.322	.322	

The units of perfusion are mL/100g tissue/min.

## Discussion

Although adequate perfusion is paramount to healing diabetic foot ulcers, the tools to quantitatively and directly assess foot perfusion are currently lacking. This pilot study assesses the feasibility of using ASL, an MRI technique without the use of intravenous contrast, to measure foot perfusion both in healthy volunteers and diabetics with neuropathic wounds. Using ASL, we measured regional foot perfusion in the healthy foot as well as peri-wound perfusion. In healthy volunteers, there was a detectable increase in perfusion with activation of intrinsic foot muscles. In diabetics with nonischemic wounds, we show that the foot is hyperemic relative to healthy volunteers at rest and that the peri-wound tissue is hyperemic compared with the tissue far from the wound.

Perfusion measurements of the plantar foot during toe flexion are in line with previously reported values, yet observed variations can be attributed to testing conditions and image processing. There was an expected increase in perfusion with sustained toe flexion, compared with rest, with the largest increase at the medial plantar foot from 30 to 64 mL/100g/min. Zheng et al similarly found that plantar foot perfusion during subjective toe flexion (volunteers were told to flex and hold) was highest on the medial aspect at 93 mL/100g/min.^18^ Edalati et al also found the greatest perfusion in the medial plantar foot during toe flexion in an MRI compatible dynamometer at 20% of their maximal effort, increasing to 17 from 9.8 mL/100g/min.^20^ Although the type of exercise used to activate intrinsic foot muscles was similar, the degree of effort was variable in these studies. The image processing protocols also differed both in creating the ROI used to average the imaging voxels and the thickness of imaging slices used to average perfusion. Lastly, the type of tissue included in the perfusion measurement also varied, as some studies included only muscle whereas our study included all tissue. The variations in results suggest that perfusion measurements using ASL require consistency in imaging protocol and conditions to obtain reproducible measurements.

In this study, we also described a quantitative peri-wound perfusion pattern that builds upon currently published qualitative perfusion patterns. We found that perfusion in the wound bed and the tissue immediately adjacent to the wound bed is hyperemic, with an approximate 50% increase in perfusion compared with the tissue far from the wound. The peri-wound perfusion pattern is in line with qualitative perfusion patterns measured by other tools, including near infrared imaging and laser speckle contrast imaging.^27^, ^28^, ^29^, ^30^ The surprising result of increased perfusion does not agree with the recently published results of peri-wound perfusion analyzed by ASL. Edalati et al found that perfusion around the wound was reduced compared with tissue far from the wound (9.5 vs 8.6 mL/100g/min).^20^ However, this group only included skeletal muscle perfusion in their ROI. In contrast, we included all tissues in the foot, including superficial soft tissue, which is the tissue perfusion primarily measured by the aforementioned qualitative tools. This specific pattern of peri-wound perfusion is known to occur in healing wounds instead of stalled non-healing wounds where the perfusion pattern indicates a hypoperfused peri-wound region compared with the remote region.^27^^,^^31^ It is likely that the perfusion profile of these patients actively undergoing wound care would reflect the perfusion pattern of healing instead of a stalled non-healing wound. However, this increased blood flow around a wound may not be sufficient to heal the wound, underlining the importance of a perfusion deficit. Furthermore, the peri-wound perfusion pattern may be drastically different in ischemic wounds. Although speculative, the pattern may show areas of hypoperfusion or areas of increased perfusion but to a degree much smaller than the increase seen in this nonischemic patient cohort. Once again, this exemplifies the concept of a limb’s perfusion deficit. Currently, the perfusion threshold that would lead to wound healing has not been quantified nor are the factors that modulate this threshold known. Although the pattern of perfusion is important, the quantification of perfusion provides more insight into the wound. Ultimately, a tool to quantify tissue perfusion will allow for the exploration of a wound’s perfusion deficit and its implications on healing.

The perfusion pattern along the wound depth may provide a more complete picture of the perfusion status of a wound. Currently, the direct assessment of tissue perfusion beyond the superficial layer is limited to indirect measures of perfusion like optical coherence tomography. Although these types of tools have been used in documenting angiogenesis at the wound edges along a tissue depth, their implications on wound healing are unknown.^30^ In this feasibility study, we showed that the peri-wound hyperemia diminishes along the tissue depth and nearly normalizes at the base of the wound. Increased wound depth has been associated with higher amputation rates and is used in wound clinical severity scores.^6^^,^^32^^,^^33^ The implication of this perfusion pattern along the wound depth and the quantitative differences between superficial and deep tissue perfusion are unknown.

There were several limitations in this feasibility study. The study group was small and recruited during a recurring wound care clinic day where there are a large number of diabetic patients with neuropathic wounds. The lack of patients with ischemic wounds limits the conclusions of this feasibility study. It is unknown whether the perfusion pattern seen in this cohort would be observed around ischemic wounds. However, imaging ischemic wounds is the next step in assessing the clinical applicability of ASL MRI. Given the results of the present study, it is likely that ASL could quantify peri-wound perfusion around ischemic wounds. Additionally, the conclusions drawn from the comparison between the healthy and diabetic groups were limited because their demographics were different. Furthermore, all the diabetic patients were male, further narrowing the applicability of our findings. The cohort demographics and lack of matching between the healthy and diabetic volunteers required study resources that were not available for this small feasibility study. Other seemingly feasible comparison groups, like using the contralateral foot in diabetics or diabetic patients without wounds as a control group, were not possible secondary to logistical constraints of this feasibility study and went beyond the study’s scope. However, these limitations present opportunities for future studies. Other features of the imaging modality that limited the study included image processing. There were small movements in the foot during the scan that we were unable to correct and may have contributed to measurement error. Lastly, the novel application of this imaging study precluded a standardized manner of imaging volunteers. Though we imaged all of the volunteers in the same fashion, there were some parameters that were not controlled such as the room temperature in the imaging suite, the volunteer’s activity prior to the scan, or avoidance of substances that may affect perfusion. The significance of these uncontrolled factors on perfusion assessed by ASL is unknown at this time.

## Conclusion

We have demonstrated the feasibility of using ASL MRI to quantify foot perfusion both in healthy volunteers and diabetic volunteers with nonischemic wounds. This imaging modality provides a noninvasive means of quantifying perfusion with standard medical equipment, rendering granular detail of foot perfusion throughout a volume of tissue. We have demonstrated that the perfusion around a nonischemic diabetic foot ulcer is 1.5 times greater than perfusion in tissue far from the wound and that the foot is hyperemic relative to the non-diabetic foot without a wound. The application of ASL in the quantitative assessment of foot perfusion is promising, yet requires larger study to elucidate its clinical implications on wound healing and the exploration of a wound’s perfusion deficit.
